# TIMP-1 downregulation modulates miR-125a-5p expression and triggers the apoptotic pathway

**DOI:** 10.18632/oncotarget.23832

**Published:** 2018-01-02

**Authors:** Sampa Ghoshal-Gupta, Ammar Kutiyanawalla, Byung Rho Lee, Juhi Ojha, Aliya Nurani, Ashis K. Mondal, Ravindra Kolhe, Amyn M. Rojiani, Mumtaz V. Rojiani

**Affiliations:** ^1^ Department of Pathology, Medical College of Georgia-Augusta University, Augusta, GA, United States of America; ^2^ Department of Medicine, Medical College of Georgia-Augusta University, Augusta, GA, United States of America

**Keywords:** TIMP-1, miR-125a-5p, NSCLC, apoptosis

## Abstract

Matrix metalloproteinases and their natural inhibitors (TIMPs) are important elements in a wide range of oncology settings. Elevated levels of tissue inhibitor of metalloproteinase-1 (TIMP-1) have often been associated with increased tumorigenesis. This has been demonstrated in a number of clinical and experimental models which include breast, gastric, colorectal and non-small cell lung carcinoma (NSCLC). Our earlier studies have identified increased angiogenic activity and aggressive tumor kinetics in TIMP-1 overexpressing H2009 lung adenocarcinoma cells. TIMP-1 overexpression has also been implicated in antiapoptotic responses, inducing a significant upregulation of Bcl-2. These TIMP-1 functions have been shown to be MMP-independent and provide insight into its pleiotropic activities. The current study examines microRNA (miRNA) interactions with this molecule. We have sought to define the relationship between TIMP-1 and miRNA by knocking down TIMP-1 in high TIMP-1 expressing lung adenocarcinoma cell lines. TIMP-1 knockdown resulted in increased expression of miR-125a-5p with a concomitant increase in apoptosis and attenuation of the tumorigenic features of these cells. We have identified TIMP-1 as a bona fide target of miR-125a-5p, and their interaction resulted in an increase in p53 expression. We further corroborated our *in vitro* data with patient samples, which exhibited an inverse correlation between TIMP-1 and miR-125a-5p expression. Our study lends support to the notion that elevated TIMP-1 levels, which are frequently associated with poor prognosis, cause aberrant modulation of miRNAs.

## INTRODUCTION

Lung cancer is clinically classified into two main histologic subtypes i.e. small cell lung cancer (SCLC) and non-small cell lung carcinoma (NSCLC). NSCLC accounts for 85% of lung carcinomas and has a 5-year survival rate of only 17%. This subtype includes large cell, squamous cell and adenocarcinomas which is the major subtype [1, 2]. Despite advances in therapy, prognosis remains dismal due to diagnosis at advanced stages [3, 4]. NSCLC represents a clinically and biologically heterogeneous group that responds differently to chemotherapeutic agents, therefore there is a dire need for identification of biomarkers that are better able to predict the response to treatment.

In recent years, tissue inhibitor of metalloproteinase-1 (TIMP-1) has emerged as an important prognostic biomarker. In several cancers including non-small cell lung cancer, high serum levels of TIMP-1 have been associated with poor prognosis [5]. Previously, we have shown that overexpressing TIMP-1 in H2009, a lung adenocarcinoma cell line resulted in aggressive tumors when implanted in the mouse brain [6]. We have also reported on the antiapoptotic activity of TIMP-1 in a lung adenocarcinoma, with delineation of the signaling pathway downstream of TIMP-1 leading to its antiapoptotic function [7].

MiRNAs are small noncoding RNA molecules that function as post-transcriptional regulators by binding mainly to the 3′ UTR of target genes. As such, they regulate gene expression either by repression of translation or degradation of mRNA [8]. It is estimated that miRNAs can bind to hundreds of different mRNAs and appear to regulate more than 50% of the protein coding genes [9]. They function either as oncomirs (when upregulated) or as tumor suppressors (when down regulated) in cancer, affecting diverse cellular processes like proliferation and apoptosis. One of the important factors hampering successful cancer therapy is drug resistance that results in failed apoptosis. TIMP-1 has been documented to play a role in drug resistance by virtue of its antiapoptotic function [10, 11]. Decreased proapoptotic miRNA or increased anti-apoptotic miRNA expression in cancer has been linked to high anti-apoptotic threshold and chemoresistance [12].

We hypothesized that the diverse functions of TIMP-1 could be attributed to its interactions with other molecules, possibly including microRNAs. Several studies have documented an involvement of miRNA in TIMP-1 function [13–15].

Hsa-miR-125a-5p has been shown in several studies to function as a tumor suppressor with repressed expression in cancerous tissue compared to adjacent normal tissue [16–18]. Hence, in the present study we have sought to determine a role for miRNA 125a-5p in two high TIMP-1 expressing NSCLC cell lines to further delineate the signaling networks at play in the diverse and sometimes paradoxical functions of TIMP-1.

## RESULTS

### Knocking down TIMP-1 increases expression of miR-125a-5p

**Figure 1:**
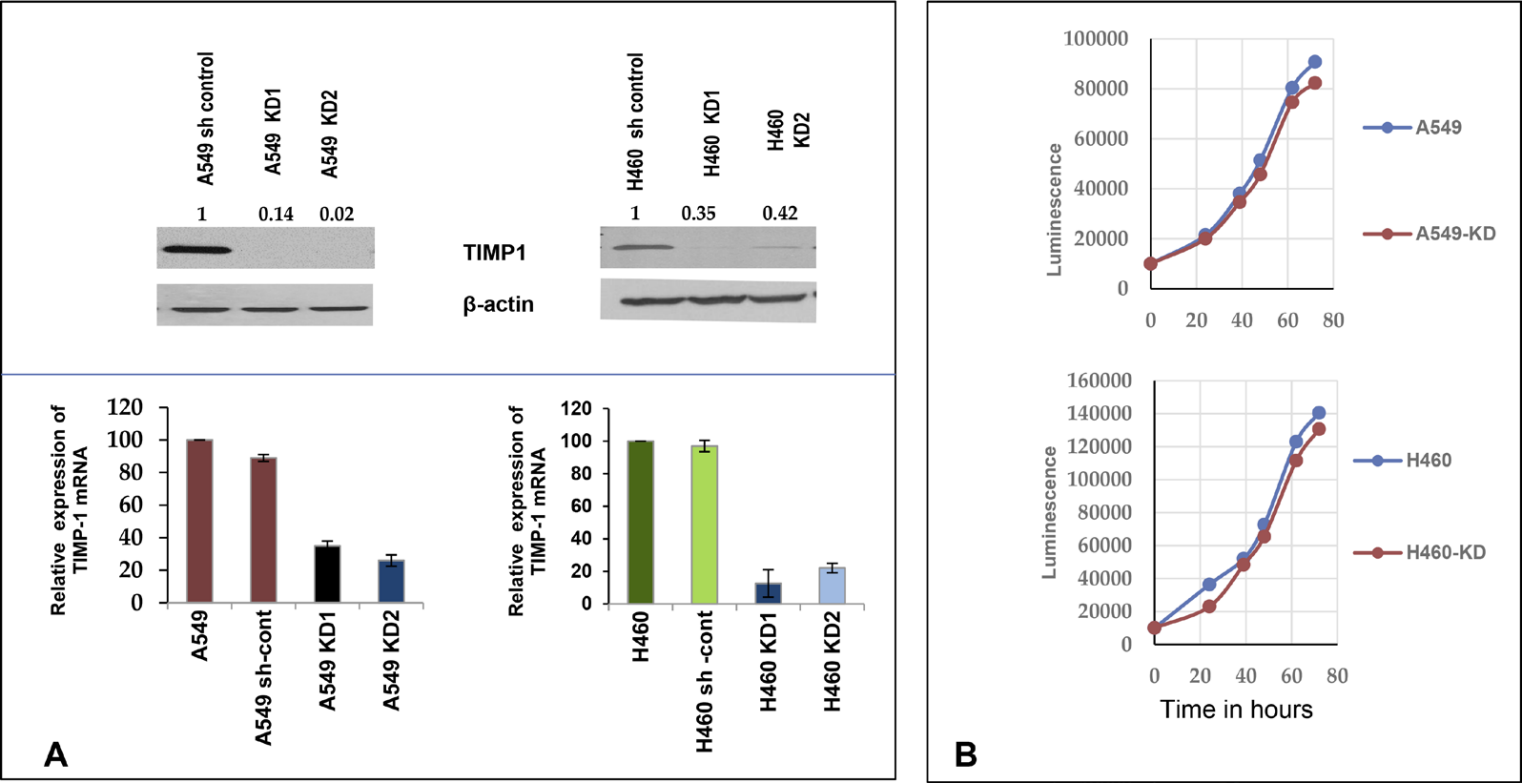

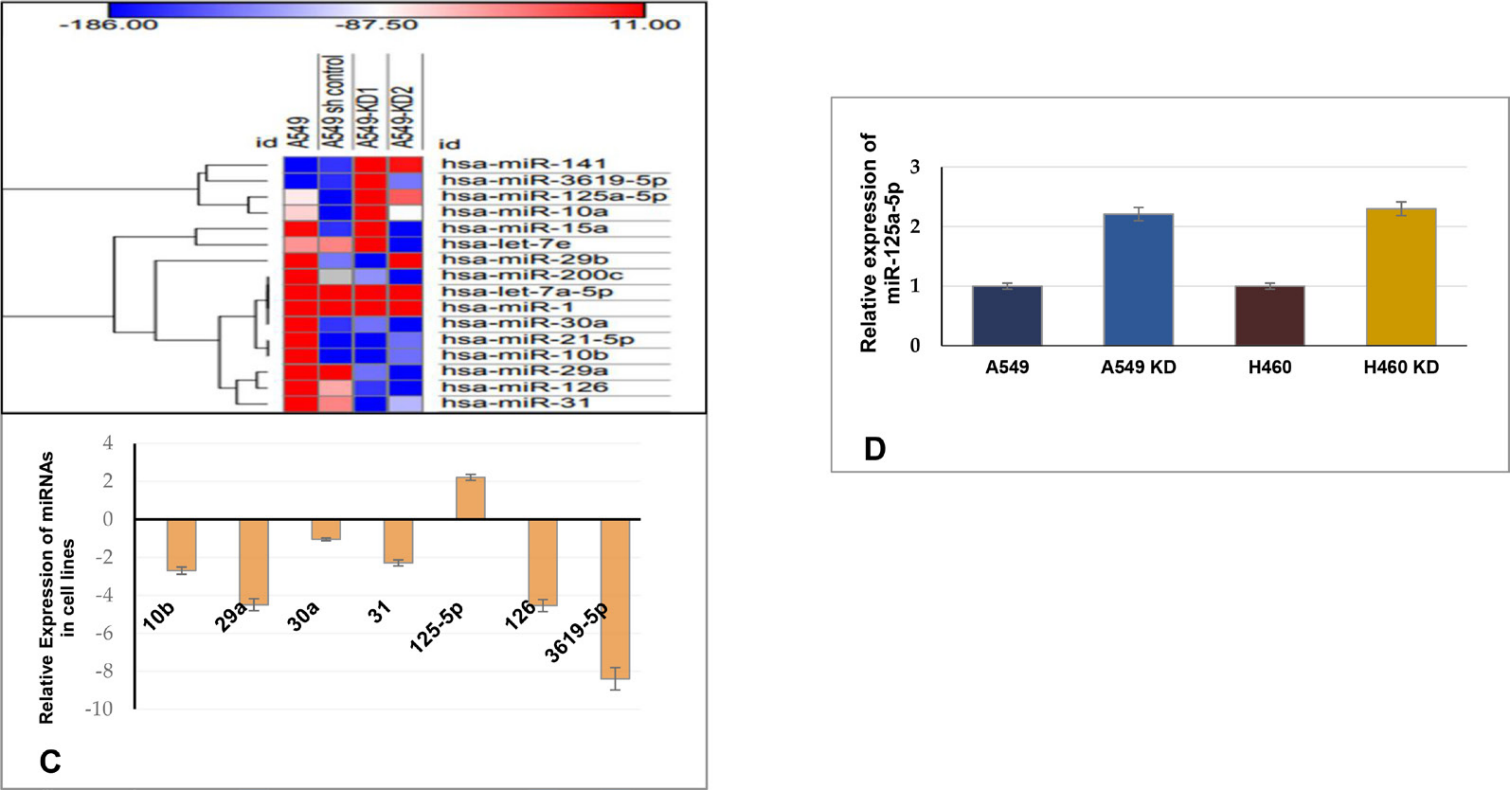

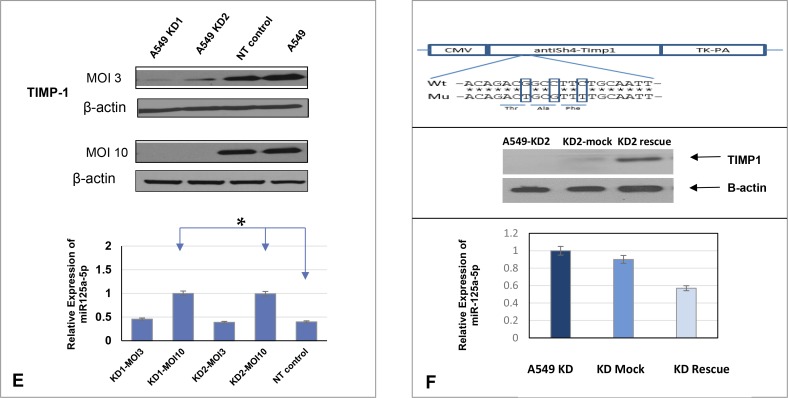
Knocking down TIMP-1 increases expression of miR-125a-5p (**A**) A549 and sh control cells with higher protein levels of TIMP-1 as demonstrated by representative western blot. The A549 TIMP-1 knockdown clones showed 80%–97% knockdown and the H460-KD clones showed about 70% knockdown of protein in an average of three experiments (top panel). Real-time polymerase chain reaction confirmed the down-regulation of TIMP-1 messenger expression. GAPDH primers were used for internal normalization with the sh controls set at 100%. The graphs are representative of average of 3 experiments ± SEM. (**B**) Cell doublings were studied in A549 and H460 and their clones; no significant changes were observed in the cell doubling times (average 18.5 hours). (**C**) Heat map of miRNA microarray (Affymetrix) expression data (fold change) in A549 and its knockdown clones was done by GENE-E (https://software.broadinstitute.org/GENE-E) through a web based tool Morpheus, showing chosen microRNA modulations. Hierarchical cluster analysis of miRNAs were done on the y axis, the legend on the right indicates miRNAs represented in the corresponding rows. The relative miRNA expression is depicted according to the color scale shown on top, red indicates upregulation and blue, downregulation. Lower panel: Expression of seven chosen miRNAs in A549 cell lines by qPCR (average of three replicates), based on microarray data. Only miR-125a-5p showed upregulation with TIMP-1 knockdown. (**D**) Confirmation of miR-125a-5p expression in A549 and H460 TIMP-1 knock-down cell lines. miR-125a-5p showed more than 2 fold upregulation with TIMP-1 knockdown; small nuclear RNA U6 was used as a reference gene to normalize the data. Figures are representation of three independent experiments ± SEM. (**E**) Differential expression of miR-125a-5p in A549 cells due to different MOI of infection by shTIMP-1 lentivirus particles. Representative western blot of TIMP-1 expression normalized to actin is shown (top panel). The bottom panel represents miRNA expression, small nuclear RNA U6 was used as a reference gene. Values are representative of 3 independent experiments (mean ± SEM; ^*^P < 0.05, Student *t* test). (**F**) Rescue experiments were performed by co-transfecting with mutated TIMP-1 plasmid. Downregulation of miR-125a-5p in the rescued cells was verified by qPCR. Topmost panel shows design of plasmid construction followed by representative western blot of TIMP-1 expression. The bottom panel shows the average expression of miR-125a-5p transcript conducted in triplicate after rescue experiment.

In order to define the modulation of microRNA profiles upon altering TIMP-1 levels, we knocked down TIMP-1 expression by shRNA in A549 and H460, two high TIMP-1 expressing NSCLC cell lines. To eliminate off-target effects, 4 independent shRNA sequences targeting different sites of TIMP-1 were utilized. We chose 2 knockdown clones for A549 (A549 KD1 and A549 KD2) and one knockdown clone for H460 (H460 KD) for our study. Both qRT-PCR and immunoblotting verified that TIMP-1 expression in A549 and H460 knockdown cells were significantly decreased compared to parental cell lines (Figure 1A). Since TIMP-1 is also known to be a mitogen, we utilized a cell proliferation assay to identify any effect of TIMP-1 knockdown on cell growth. We found that these manipulations had no effect on cell proliferation (Figure 1B).

To establish a role for microRNA in the pleotropic functions of TIMP-1, we carried out GeneChip microRNA array analysis. GeneChip^®^ miRNA 2.0 microarrays from Affymetrix^®^ were used to examine the expression of microRNAs in total RNA extracted from A549 cells and its knockdown clones. Of the differentially expressed miRNAs, we selected 7 miRNAs that have previously been implicated in NSCLC and then further verified our array results by qPCR (Figure 1C). We also verified our data by RT^2^ profiler PCR arrays and identified miR-125a-5p as an upregulated miRNA (data not shown) in the knockdown cells.

We observed that knocking down TIMP-1 increased the endogenous expression of miR-125a-5p by more than two-fold compared to 6 other miRNAs subjected to qPCR. Since miR-125a-5p showed a significant up-regulation with TIMP-1 knockdown and since it has previously been reported to be both pro-apoptotic and down-regulated in lung cancer, we focused our further studies to miR-125a-5p (Figure 1D).

To validate that TIMP-1 down-regulation was causing an increase in miR-125a-5p levels, we infected the cells with different multiplicity of infection (MOI) of sh-TIMP-1 lentivirus or a non-targeted control. As shown in Figure 1E, MOI of 3 does not downregulate TIMP-1 as effectively as an MOI of 10 (upper panel). This is reflected in the lesser increase in the level of miR-125a-5p at an MOI of 3 compared to an MOI of 10 (lower panel).

To further confirm bona fide knockdown of TIMP-1, we carried out a rescue assay for the A549 KD2. This resulted in the re-expression of TIMP-1 and a subsequent decrease in miR-125a-5p expression (Figure 1F).

### MiR-125a-5p upregulation mitigates tumorigenic features and directly targets TIMP-1

**Figure 2:**
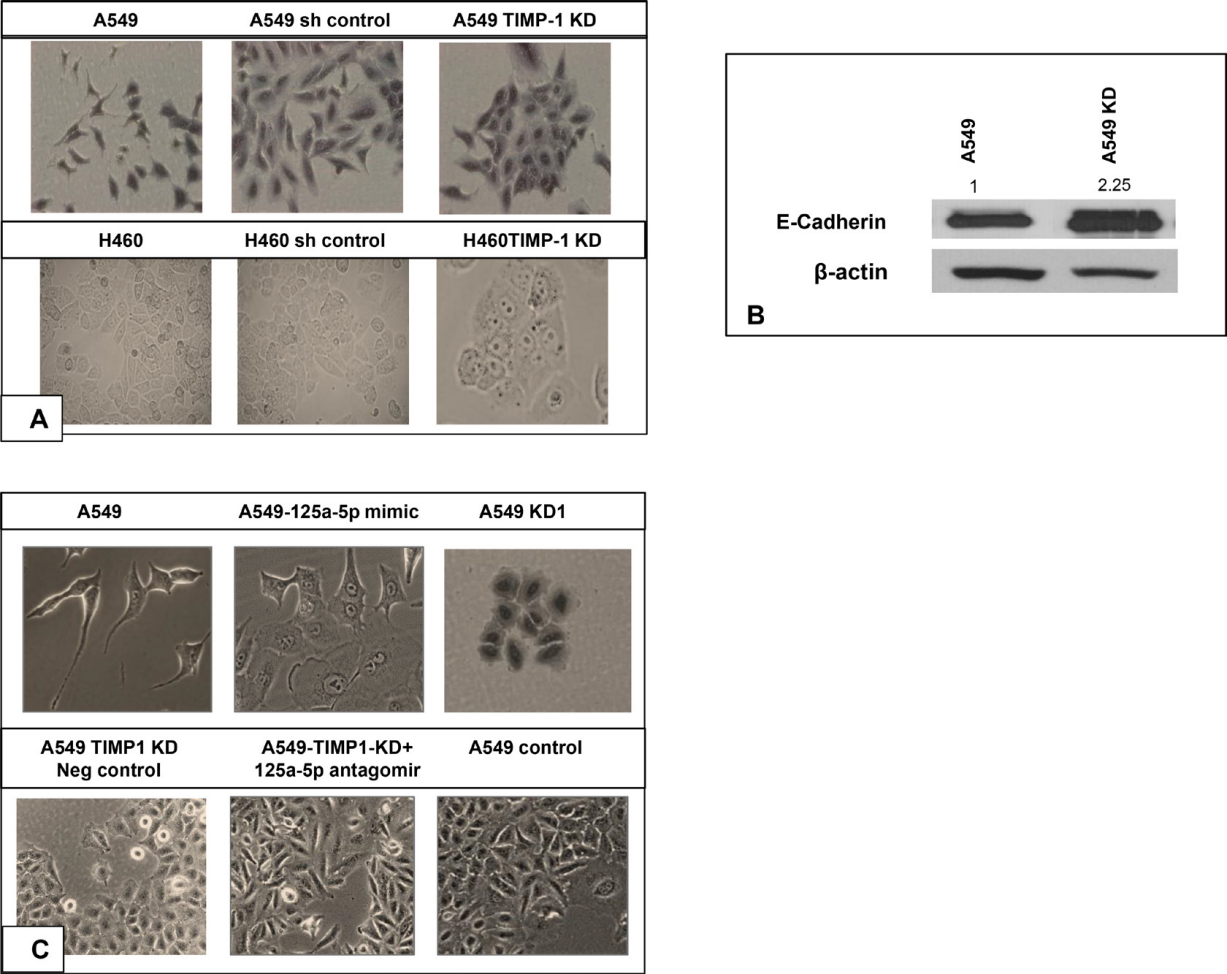

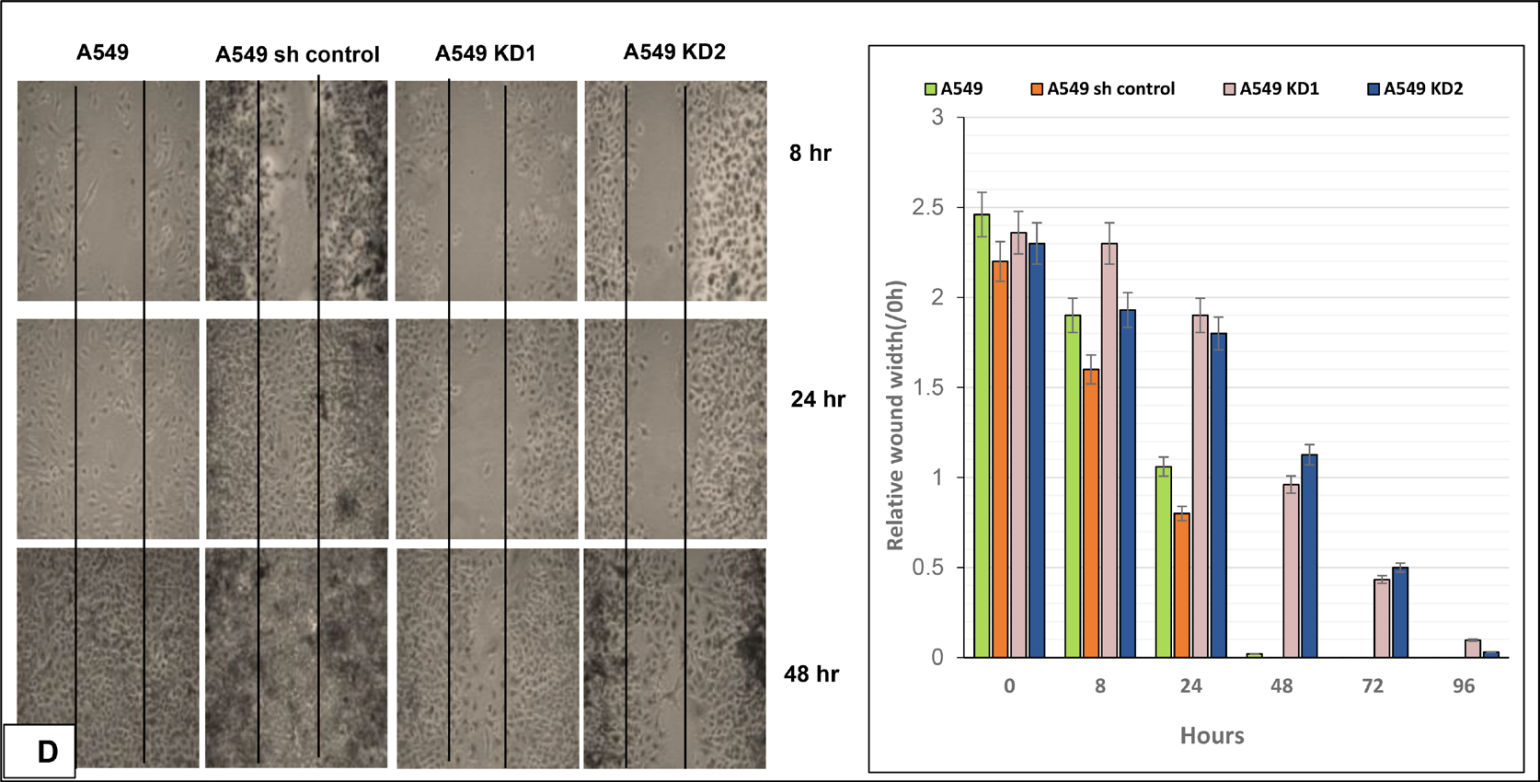

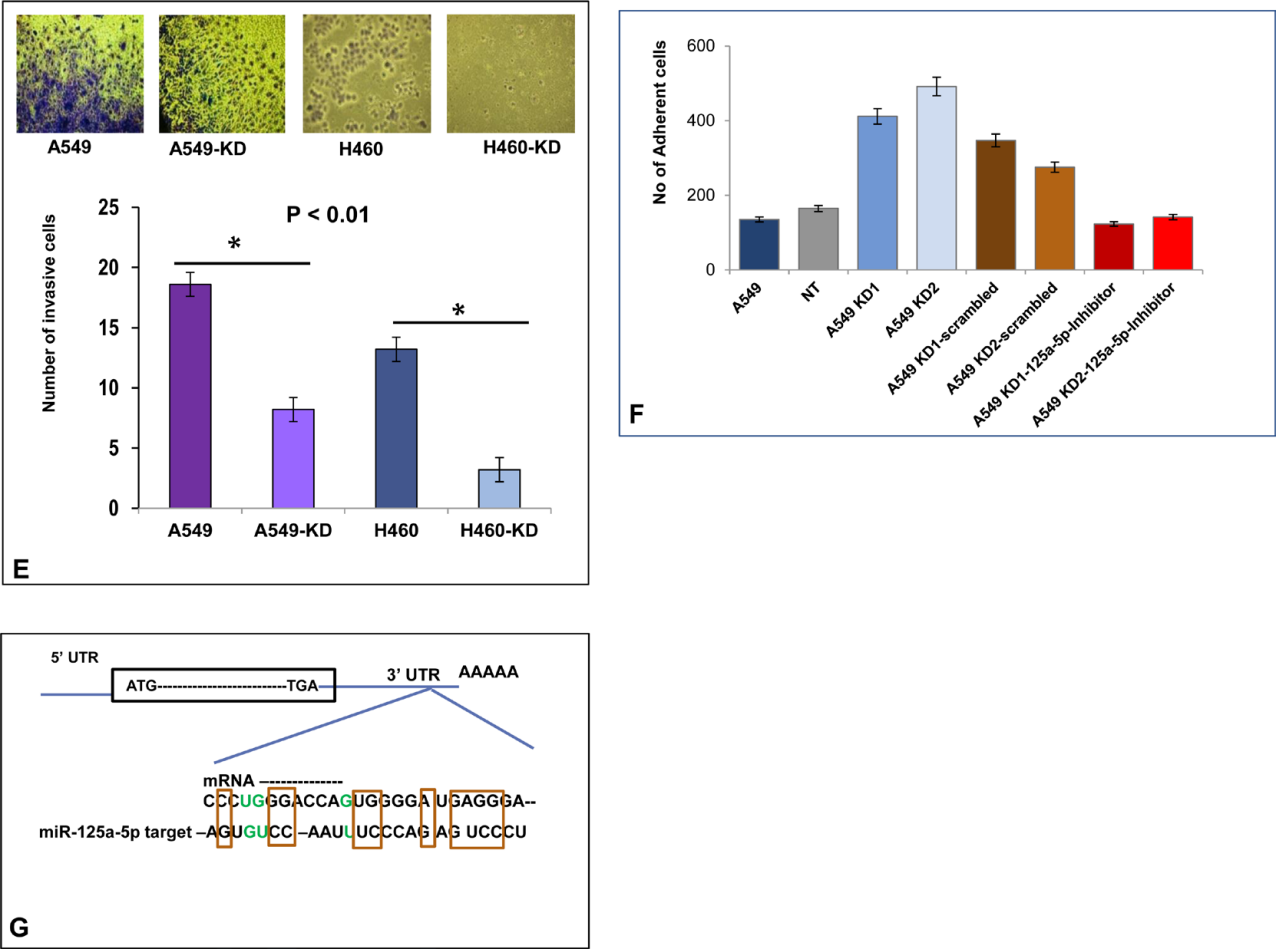

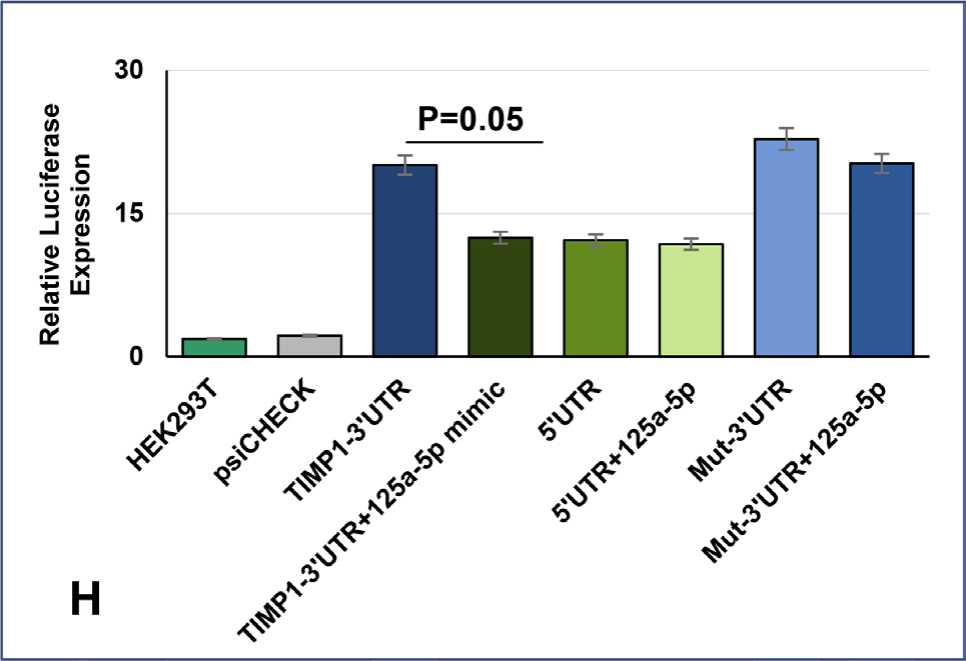
Loss of EMT and tumorigenic features following TIMP-1 knockdown (**A**) Both A549 and H460 cells changed from an initial spindled shape to a more cohesive epithelioid morphology. (both panels are at 20× magnification). (**B**) Representative figure showing higher expression of E-cadherin in A549 knockdown cells by western blot. The data was normalized to actin, the values represent average of 3 independent experiments. (**C**) Introduction of miR-125a-5p mimic in the normally spindled A549 cells converts them to a more epithelioid appearance. Similar epithelioid change is seen in the A549 KD clone. Top figures are at 40X magnification and bottom figures are at 20X magnification). Addition of 125a-5p antagomirs to TIMP-1 KD clones converted the cell morphology from epithelioid to mesenchymal type, similar to the control A549 cells. (**D**) Impact of TIMP-1 downregulation on cell migration *in vitro* using wound assay: Black lines indicate the wound borders at the beginning of the assay and were recorded at 0 (not shown in picture), 8, 24 and 48 hours post scratch. Relative scratch gap was calculated as the ratio of the remaining scratch gap at the given time point and the original gap at 0 hours. Bar graphs represent average value of three replicates ± SEM. (**E**) TIMP-1 knockdown in A549 and H460 results in less invasive cells compared to wild type A549 and H460. [*p* < 0.01 (one way ANOVA)]. The data represents mean ± SD (*n* = 2). (**F**) The A549 KD clones showed less adhesion with the introduction of miR-125a-5p inhibitors. The data represents mean ± SD (*n* = 3). (**G**) MiR-125a-5p is a bona fide target of TIMP-1: Sequence alignment of 3′UTR region of TIMP-1 mRNA and miR-125a-5p. Analysis with RNA22 and BLASTN revealed conserved critical nucleotides that may serve as legitimate TIMP-1 target. GU wobbles are indicated in green. (**H**) Luciferase activity was measured 48 h post-transfection in HEK293T cells, with miR-125a-5p mimics and reporter plasmids containing wild type or mutated TIMP-1 3′-UTR, normalized to activity in cells transfected with plasmid only control. Significant reduction in luciferase activity was observed with the introduction of mimics but not with mutated TIMP-1 3′UTR. The data represent the mean ± SEM. ^*^P = 0.05, *n* = 2.

Upon knocking down TIMP-1 in A549 and H460, the cells manifest interesting changes in morphologic appearance. High TIMP-1 expressing A549 cells showed spindle-shaped, fibroblast-like morphology and increased cell scattering, both of which may be associated with an EMT-like phenomenon whereas TIMP-1 knockdown cells displayed cobblestone-like epithelioid morphology and a closely adherent arrangement. Similar profiles were observed with H460 cells (Figure 2A).

This observation appeared to be in tune with the aggressive and tumorigenic potential of high TIMP-1 expressing cells. Recent studies have also demonstrated that TIMP-1 overexpression causes EMT like changes in cell morphology and gene expression [19]. We confirmed the loss of EMT-like phenotype by western blot for E-cadherin, the epithelial marker typically lost during EMT. Figure 2B shows E-cadherin upregulation in TIMP-1 KD clone of A549.

We hypothesized that this morphologic alteration following TIMP-1 knockdown may be associated with miR-125a-5p interactions. To test this hypothesis, we transfected mimics of miR-125a-5p into A549 cells and observed that there was a morphologic change whereby the cells appeared more epithelioid i.e. an appearance similar to the morphology of A549 KD cells. In contradistinction, introducing antagomirs into the A549 KD clone reverted the morphology from epithelioid to mesenchymal patterns (Figure 2C).

Thus morphologic alterations could be induced with either TIMP-1 KD or miR-125a-5p overexpression.

To determine if the more epithelial morphology was associated with reduced tumorigenic behavior, we carried out the following functional assays. A wound healing assay showed that TIMP-1 KD clones were less migratory compared to parental cells (Figure 2D). In addition, a cell invasion assay exhibited decreased invasion by the KD clones when compared to A549 and H460 parental cell lines (Figure 2E). Finally, invasive behavior is often associated with reduced adherence [20, 21], we therefore performed an adhesion assay. We determined that A549 KD cells are more adherent on a coat of Matrigel compared to A549, and KD clones with added antagomirs behave similar to parental A549 cells (Figure 2F).

The above studies indicated that TIMP-1 might be a target gene of miR-125a-5p, therefore, different computer-generated algorithms including RNA 22 and miRcode were utilized to identify potential seed sequences in the TIMP-1 gene. Analysis with BLASTN revealed that the TIMP-1-3’UTR contained conserved critical nucleotides that may serve as legitimate targets of miR-125a-5p (Figure 2G).

Both the 3′ and the 5′ UTR sequences and their mutant motifs were cloned into luciferase reporter vector and transfected into HEK-293T cells. MiR-125a-5p mimics repressed the luciferase activity in the 3′ UTR constructs and no change was seen in the 5′ UTR constructs. Mutant 3′ UTR failed to inhibit luciferase activity despite the presence of miR-125a-5p mimics (Figure 2H).

These results collectively, demonstrated that TIMP-1 is indeed a *bona fide* target of miR-125a-5p.

### Decreased miR-125a-5p expression in a subset of NSCLC tissues

**Figure 3:**
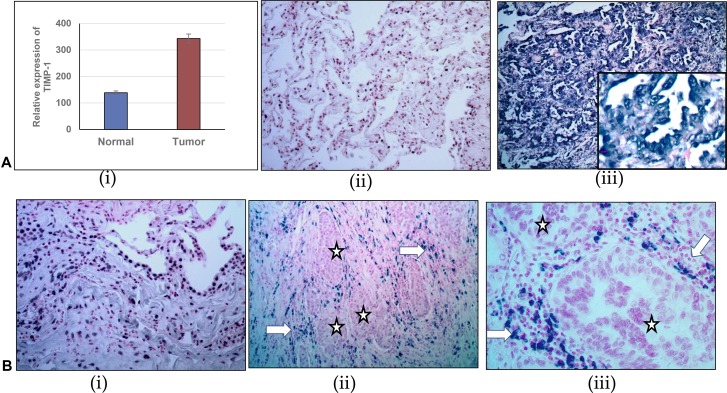

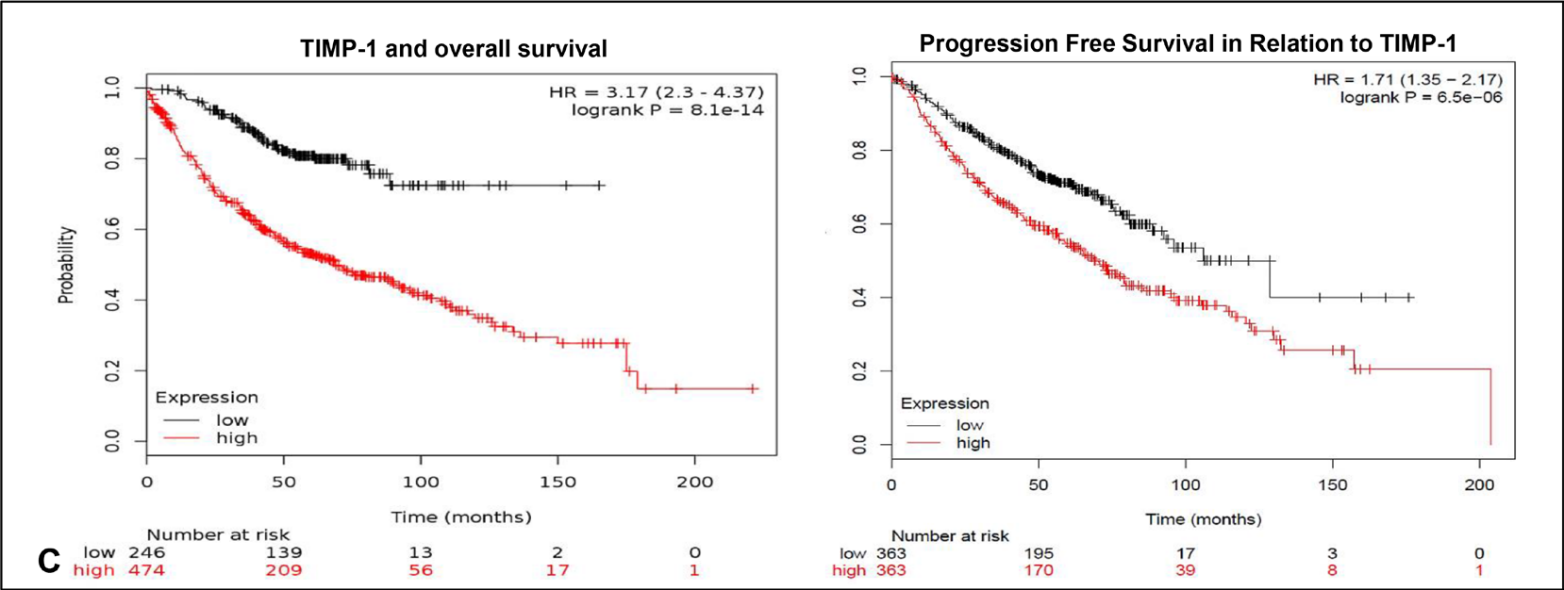
Comparison of relative expression of TIMP-1 and miR-125a-5p in normal adjacent lung tissue and lung adenocarcinoma (**A)** (i) Relative expression of TIMP-1 as detected by RT-PCR. (ii) and (iii) TIMP-1 expression detected by *in-situ* hybridization: (ii) Minimal TIMP-1 expression in normal adjacent tissue, 40× original magnification; (iii) High expression of TIMP-1 in representative tumor tissues 100× original magnification, inset- 400× original magnification. (**B)** (i): miR-125a-5p expression in normal adjacent tissue, detected by *in-situ* hybridization: 100× original magnification; (ii) and (iii) Almost total absence of miR-125a-5p expression in tumor cells (^*^) although some expression is seen within surrounding stromal region of the tumor (arrows) - 100× and 400× original magnification, respectively. (**C**) Kaplan Meier plots showing survival of patients in relation to TIMP-1 expression: To show the relationship of TIMP-1 with survival of patients we curated data manually, from expression arrays by KM Plotter, reference: PLoS One. 2013 Dec 18;8(12):e82241.54,675 Affymetrix probe set IDs (Affymetrix HG-U133A, HG-U133 Plus 2.0 and HG-U133A 2.0) and 70,632 gene symbols.

TIMP-1 has been associated with different solid tumor progression but its clinical significance in lung adenocarcinoma remains inadequately explored. Our initial observations prompted us to further investigate the significance of this relationship between TIMP-1 and miR-125a-5p in human tumor samples derived from wedge biopsies or surgical resections. With appropriate IRB approval, the Surgical Pathology records at Augusta University Medical Center were searched for consecutive cases of surgically resected lung cancer with a histologic diagnosis of NSCLC/adenocarcinoma. We first determined the expression level of TIMP-1 by RT-PCR in 10 such tumors and paired normal adjacent tissues (NAT). When compared with NATs, the expression levels of TIMP-1 were significantly upregulated in adenocarcinoma tissues (paired *T*-test, *P* = 0.03, *t* = −2.3), as shown in Figure 3A (i).

We then divided the samples into two groups, low and high TIMP-1 expressing tumors. Comparing malignant and normal groups, we used the criterion of a 2 ^–DDCt^ value change of less than 2 between them as low, and more than 2 being high. We found that 60% of tumors (*n* = 6/10) showed increased levels of TIMP-1. Additionally, endogenous expression of TIMP-1 and miR-125a-5p in the tumors was detected with commercially available LNA-modified DIG-labeled probes. Morphologically, we found that there was an inverse relationship between TIMP-1 and miR-125a-5p expression with 70% (*n* = 7/10 tumors showing a negative correlation. TIMP-1 distribution was predominantly high within tumor cells and stroma, compared to normal adjacent lung tissue, Figure 3A (ii) and (iii). Although Figure 3B (i) reveals significant expression of miR-125a-5p in normal adjacent lung tissue, we noted that there was an almost total absence of miR-125a-5p expression in tumor cells. There is however localization of this miRNA within the stroma as illustrated in Figure 3B (ii) and (iii).

Data extracted from the public database was plotted to generate Kaplan Meier curve using KM Plotter (Figure 3C). TIMP-1 levels correlated with shorter overall survival in patients with lung adenocarcinoma [log rank *P* = 8.1e-14, with a hazard ratio (HR) of 3.17 (2.3–4.37)]. A significant correlation was also indicated in progression free survival, with a log rank *p* value = 6.5e-06; HR = 1.71 (1.35–2.17). Together these results further support the contention that down regulated TIMP-1 contributes to better prognosis of NSCLC patients, probably by the upregulation of miR-125a-5p.

### miR-125a-5p induces apoptosis and affects TIMP-1 via a signaling mechanism involving p53

**Figure 4:**
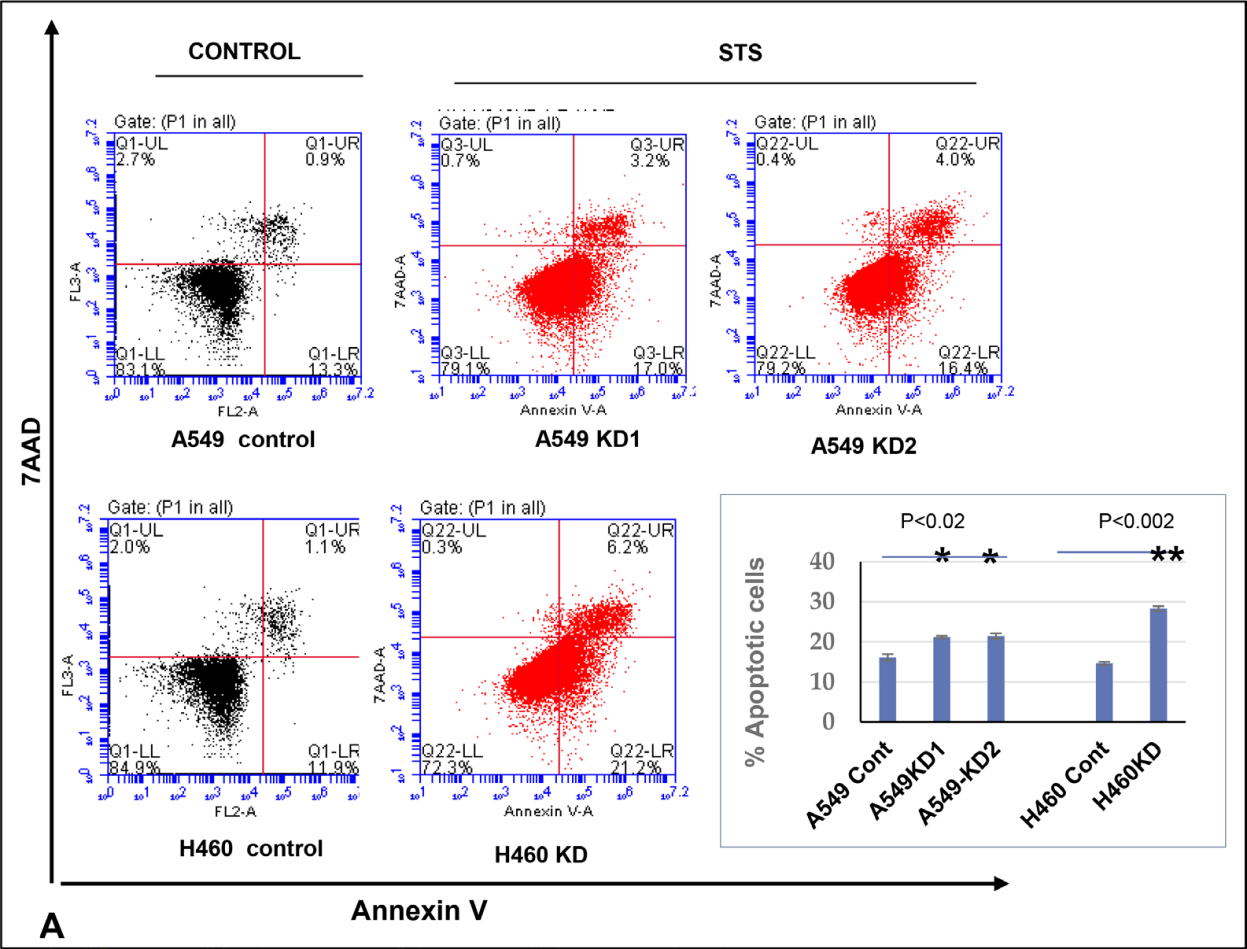

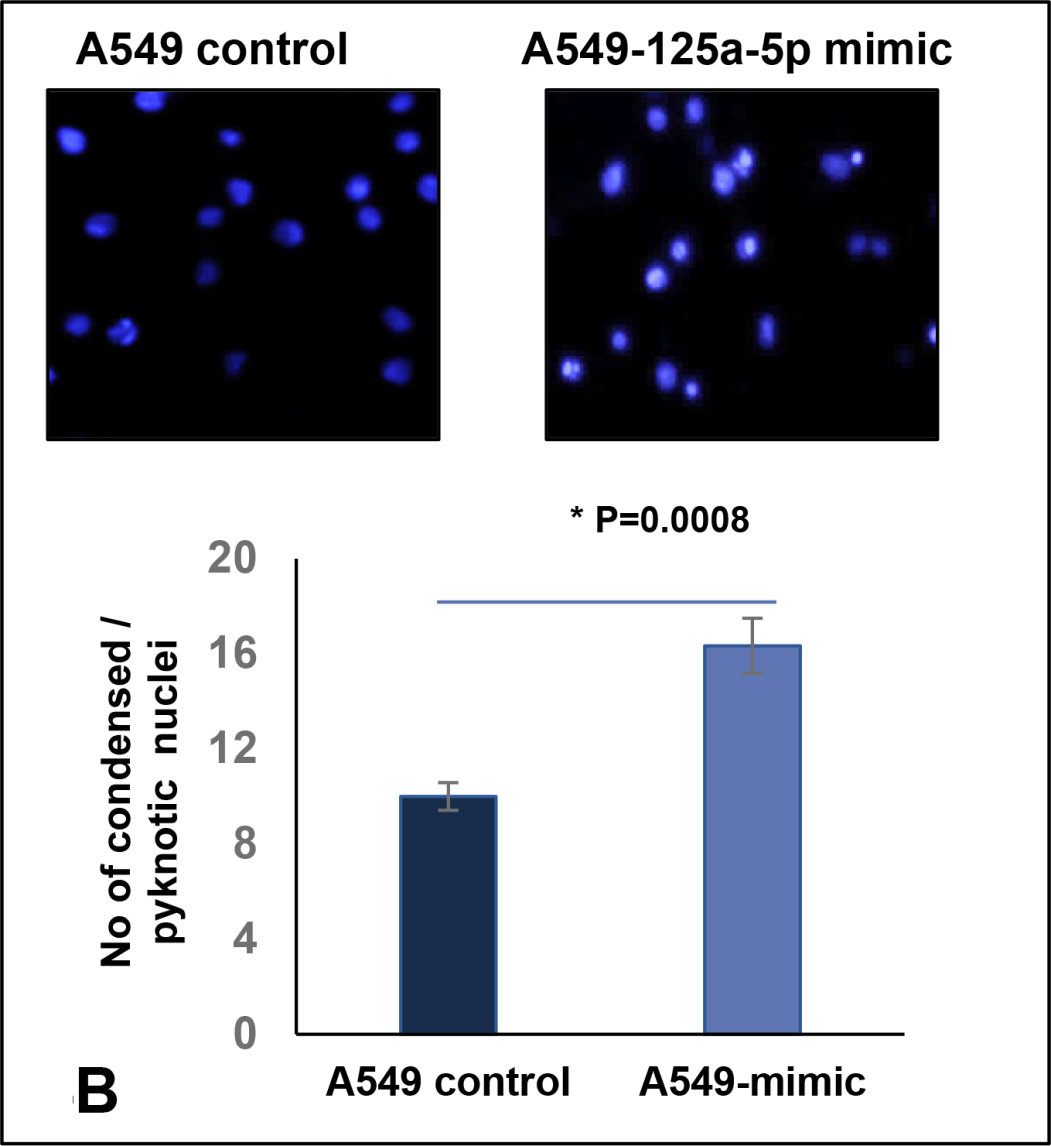

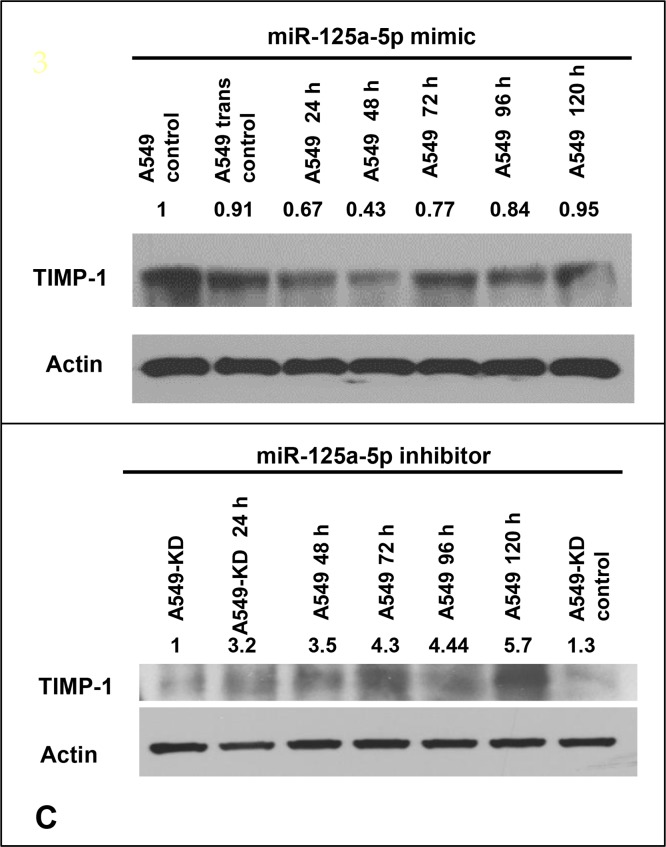

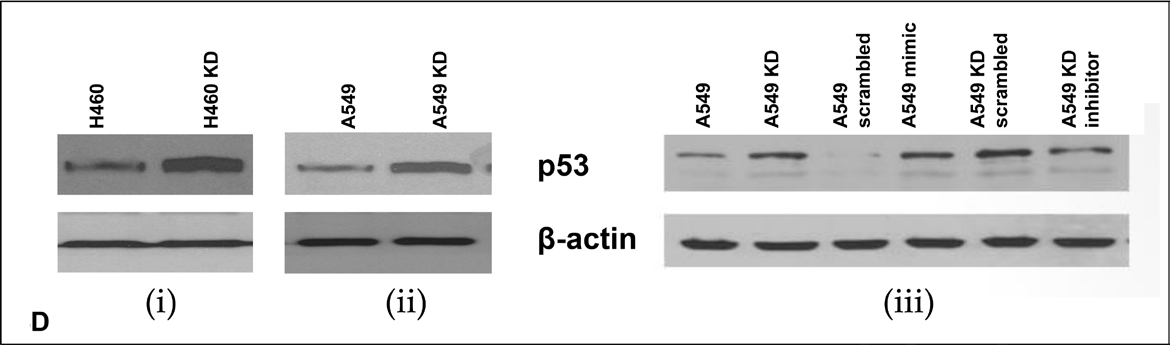

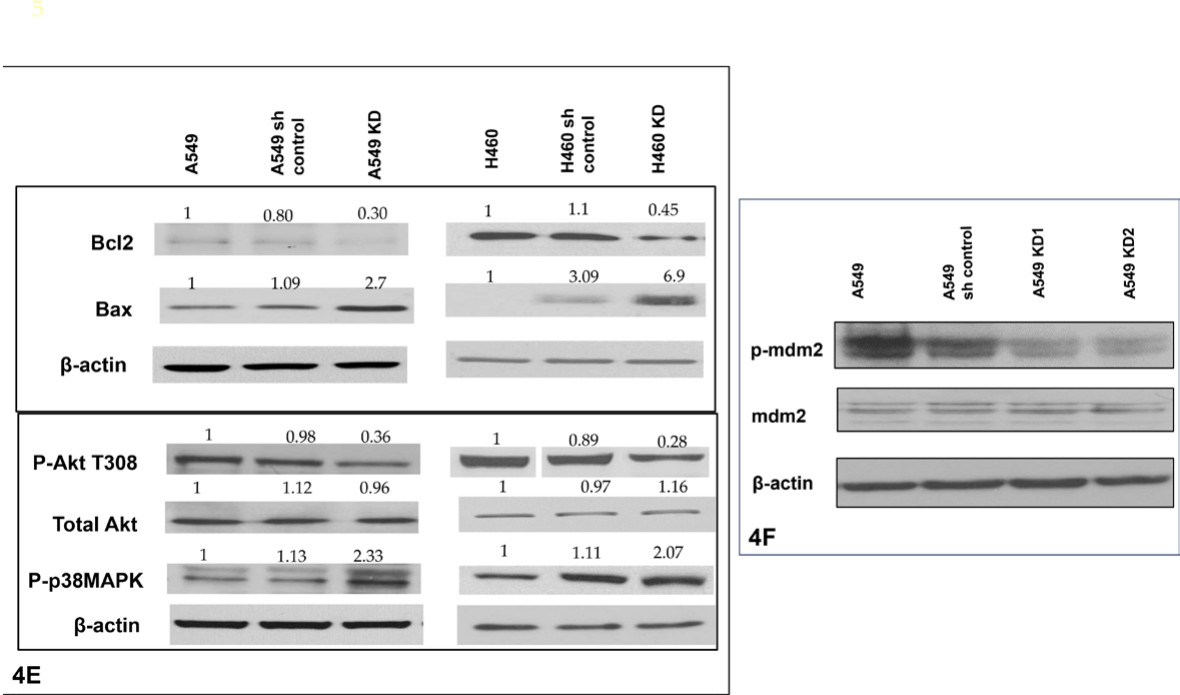
Increased level of miR-125a-5p in TIMP-1 KD clones is associated with induction of apoptosis and directly targets TIMP-1 (**A**) Flow cytometric analysis of A549 and H460 cells treated with 0.5 μM Staurosporine for 1 hour and stained with Annexin V-PE and 7AAD. Representative figures showing more apoptosis or cell death seen in TIMP-1 KD cells. Lower Right quadrant-Annexin positive cells, showing early apoptosis; Upper Right quadrant-Annexin positive, showing late apoptosis; Upper Left quadrant showing dead cells. Only live cells were considered for analysis (P1). Graphical representation of the data (% apoptotic cells ± SEM) from two independent experiments is shown on the right. (*p* < 0.02^*^, *p* < 0.002^**^, *t*-test). (**B**) A549 cells were transfected with miR-125a-5p mimic and stained with Hoechst 33342. MiR-125a-5p cells showed significant increase in pyknotic nuclei as represented in the figure and graph below (*p* = 0.0008). The graph represents average value of 3 experiments ± SD. (**C**) A549 cells were transfected with miR-125a-5p mimic (10 nM) followed by western blot analysis to determine the level of TIMP-1 protein. The cells were harvested at 24, 48, 72, 96 and 120 hours with maximum TIMP-1 downregulation at 48 hours (top). Introduction of miR-125a-5p mimic induced downregulation of TIMP-1 after 48 hours in A549 cells. Representative figure showing upregulation of TIMP-1 with addition of miR-125a-5p antagomirs (5 nM) increased the expression of TIMP-1 level over 5 days. (**D**) Upregulation of p53 in A549 cells after TIMP-1 knockdown: Transfection of miR-125a-5p mimic upregulates p53 and inhibitors of miR-125a-5p downregulates p53 in A549 cells. (**E**) Status of pro and antiapoptotic proteins: With TIMP-1 knockdown the anti-apoptotic proteins phospho-Akt and Bcl2 levels were down-regulated and the prp-apoptotic proteins Bax and phospho-p38MAPK were upregulated. The protein expression was normalized to β-actin. Data is representative of three independent experiments. (**F**) Representative protein levels of mdm2: mdm2 phosphorylation is reduced after TIMP-1 knockdown, the total protein level remains unaltered.

Although miR-125a-5p functions as an oncogene in some cancers, in most cancers it has been documented to have a tumor suppressor function. In the latter scenarios, it functions primarily by inducing apoptosis. Additionally, TIMP-1 has a well-documented anti-apoptotic function. Therefore we induced apoptosis using Staurosporine and analyzed cell death using Annexin V. Dead cells were excluded by 7AAD. We found that TIMP-1 KD clones were significantly more apoptotic compared to the parental A549 cells as shown in the images and graphical representation in Figure 4A.

Since our postulate was that the increased apoptosis seen in KD clones was occurring via miR-125a-5p up-regulation, we transfected mimics of miR-125a-5p into parental A549 cells which resulted in increased apoptosis as measured by Hoechst assay where a higher number of pyknotic nuclei are seen with the addition of mimics (Figure 4B top panel).

The increase in apoptotic nuclei could be well correlated with TIMP-1 levels. Figure 4C shows that upon addition of mimics to parental A549, there is a significant decrease in TIMP-1 levels on day 1 and day 2 with an increase from day 3 onwards. Alternatively, adding antagomirs of miR-125a-5p to KD clones shows a consistent increase in TIMP-1 levels over a 5-day period (Figure 4C, bottom panel).

A role for p53 or an association with miR-125a-5p has been identified in several earlier studies. As a first step in attempting to elucidate the signaling pathway involved in apoptosis, we investigated whether expression of p53 was altered upon knocking down TIMP-1. Our experiments revealed that the protein level of p53 is indeed increased in A549 KD and H460 KD clones (Figure 4D, (i) and (ii) respectively). We also defined a similar increase in p53 level by addition of miR-125a-5p mimics to A549 cells as seen in the right panel of Figure 4D, (iii). Additionally, introduction of antagomirs of miR-125a-5p into A549 KD clones by transfection resulted in a decrease in p53 level (Figure 4D, iii) thus indicating that miR-125a-5p was indeed working through p53 in bringing about apoptosis. Apoptotic activity was confirmed by showing a decrease in Bcl-2 levels and a concomitant increase in the protein levels of Bax (Figure 4E).

The cytoplasmic pool of p53 in cells is modulated by the phosphorylation of Akt, the active Akt then phosphorylates mdm2 and stabilizes it [22]. Mdm2 is responsible for the ubiquitination and degradation of p53. We therefore investigated phospho-Akt levels in KD clones compared to parental A549 and H460. Phospho-Akt level was markedly decreased in TIMP-1 KD clones of both A549 and H460 (Figure 4E). We also identified an increase in p38 MAPK phosphorylation which has been documented to be proapoptotic. Finally, mdm2 phosphorylation was also found decreased in the TIMP-1 KD clones of A549 compared to parental cells (Figure 4F). Thus we conclude that TIMP-1 KD upregulates hsa-miR-125a-5p and induces apoptosis via mdm2 and Akt mediated pathways.

## DISCUSSION

Lung cancer is the leading cause of cancer deaths globally with one in four cancer deaths occurring from lung cancer [23]. For multiple cancers, it has been shown that high TIMP-1 levels in blood are associated with unfavorable prognosis and a decrease in progression-free survival [5, 24] yet it has not been established as a viable biomarker for cancer. Although classically identified as an endogenous inhibitor of MMPs and hence an inhibitor of tumor growth, many studies over the years have documented its paradoxical tumor promoting role in apoptosis, angiogenesis, proliferation and tumor metastasis. In recent years, it has been also established that TIMP-1 has a role in epithelial to mesenchymal transition (EMT) [25] premetastatic niche formation [26] and interaction with cancer - associated fibroblasts [27].

Our earlier studies have shown that over-expressing TIMP-1 in NSCLC cell line H2009, resulted in more aggressive and vascular tumors in mice compared to controls. These *in vitro* and *in vivo* investigations provided insight into the wide range of activities that have been attributed as MMP-independent functions of TIMP-1 [6]. Subsequently, we have examined interactions of TIMP-1 in the context of its antiapoptotic actions. We showed that TIMP-1 overexpression resulted in significant upregulation of Bcl-2 and yielded well-defined antiapoptotic activity. Additionally, a co-dependency of TIMP-1 and Bcl-2 RNA and protein levels was identified, such that abrogating Bcl-2 caused a downregulation of TIMP-1 but not TIMP-2 [7]. These results contributed to a better understanding of the multiple functions of TIMP-1 and its role as a potential biomarker.

In the present study we have sought to identify the epigenetic role of miR-125a-5p in the pleiotropic functions of TIMP-1 in lung cancer. Our study puts forward the notion that high TIMP-1 levels, associated with various cancers, modulates miRNA expression. Aberrant patterns of miRNA expression have been correlated with many different malignancies [28]. Additionally, miR-125a-5p has been shown to be modulated in several cancers including lung cancer and in most cases has been documented to be a tumor suppressor as its expression is down regulated in tumor tissue compared to adjacent healthy tissue [16, 29–31]. As such, it has been shown to inhibit cell proliferation and induce apoptosis [32, 33].

Our data has shown that knocking down TIMP-1 in two high TIMP-1 producing NSCLC cell lines A549 and H460, resulted in significant up-regulation of hsa-miR-125a-5p.

It is intriguing that TIMP-1 knockdown clones exhibited a cobblestone-like epithelioid morphology compared to parental A549 and H460 cells. Moreover, adding miR-125a-5p mimics to parental cell lines also resulted in epithelioid morphology. We confirmed this loss of EMT feature by determining elevated E-cadherin levels in A549 KD clones. Additional confirmation is provided by the observation that A549 KD clone reverted back to spindle shape i.e. mesenchymal profile, upon adding 125a-5p antagomirs.

Recent studies have demonstrated a role for TIMP-1 in EMT, a feature associated with aggressive migratory cancer cells [34]. Our functional assays corroborate these results, showing that knocking down TIMP-1 makes the cells less invasive and less migratory in Boyden chamber and wound healing assays respectively. TIMP-1 KD clones were also more adherent in an adhesion assay confirming the tumor-promoting function of TIMP-1.

To translate these observations found in several studies into the clinical environment, we obtained patient samples of lung adenocarcinoma tissues. Formalin-fixed paraffin embedded lung adenocarcinoma tissue and adjacent normal tissue samples were subjected to *in situ* expression detection of miR-125a-5p and TIMP-1 by ISH (*in situ* hybridization). Samples with low expression of miR-125a-5p could be correlated with high TIMP-1 mRNA expression in the majority of tumors examined, thus confirming our postulate that TIMP-1 levels and miR-125a-5p levels are inversely correlated. We also confirmed that TIMP-1 is a bona fide target gene of miR-125a-5p.

TIMP-1 is an anti-apoptotic molecule as shown by us and others [7, 35]. Interestingly miR-125a-5p is proapoptotic in systems where it exhibits a tumor suppressor function. Thus the KD clones showed increased apoptosis as determined by Annexin V assay upon induction with Staurosporine, although we did not see any change in cell proliferation (see above).

In order to determine the mechanism by which miR-125a-5p brings about apoptosis we first determined the level of p53. Few studies that have documented the tumor suppressor or the oncomir function of miR-125a-5p have demonstrated regulation of p53. In the context of oncomir function, miR-125a-5p binds to the 3′ UTR of p53 thus suppressing its expression [36, 37]. In its tumor suppressor role, it is postulated that miR-125a-5p indirectly upregulates p53 [29, 38]. We thus determined whether p53 was affected upon up-regulation of miR-125a-5p and showed by western blot that its level is indeed increased upon knocking down TIMP-1. This observation is not without precedent, as other studies have also shown involvement of p53 in TIMP-1 modulation. Rossi *et al.* showed that p53 levels increased in TIMP-1^−/−^ hematopoietic stem cells [39] and Zhang *et al.* [40] found that knocking down TIMP-1 inhibited cell proliferation and arrested cell cycle via upregulation of p53 in adipose stem cells.

P53 is considered the master regulator of apoptosis. The classical documented function of p53 is as a transcription factor that controls the expression of a large cohort of genes under stress conditions [41]. However, over the years, transcription-independent apoptotic functions of p53 have surfaced documenting its role at the mitochondrial membrane and its interaction with pro and anti- apoptotic proteins to induce mitochondrial outer membrane permeabilization (MOMP) as reviewed in [42].

It is possible that there may not be any direct interaction between TIMP-1 and p53 since sh KD of p53 did not affect TIMP-1 levels (data not shown). It is therefore likely that alteration in p53 levels in our study may simply be a downstream effect.

Several studies have documented the pro-apoptotic role of miR-125a-5p to be occurring through p53. Studies including ours have shown that TIMP-1 and Bcl-2 levels are interdependent [7, 43]. Also, it has been documented that the overexpression of Bcl-2 or Bcl-XL inhibits mitochondrial p53 accumulation and apoptosis [44]. Hence it is probable that a decrease in Bcl-2 levels upon TIMP-1 downregulation affects p53 levels and apoptosis.

Furthermore TIMP-1 and p53 protein levels could be manipulated by using mimics and antagomirs of miR-125a-5p. We defined the mechanism behind this modulation by dissecting the pathway involved in TIMP-1 effects on p53. We found that knocking down TIMP-1 resulted in inhibition of Akt activity via its dephosphorylation. Multiple studies have documented survival and proliferative effects of TIMP-1 and Bcl-2 to occur via Akt [45–48]. Also, it has been shown that miR-125a inhibits invasion by suppression of PI3K/Akt pathway [49]. Furthermore, activated Akt has been shown to attenuate mitochondrial p53 accumulation [50], phosphorylate BAD and inhibit p38 and apoptosis [51]. Non-phosphorylated Akt is unable to activate mdm2, a prerequisite for p53 ubiquitination and degradation [52]. Mdm2 can ubiquitinate both the nuclear and the cytoplasmic p53 [53].

In summary, we have shown that knocking down TIMP-1 in NSCLC cell lines resulted in increased apoptosis with concomitant upregulation of miR-125a-5p which could be acting via epigenetic mechanisms. Adding mimics of 125a-5p to parental cell lines decreased TIMP-1 levels and increased apoptosis. We were able to correlate *in vitro* data with patient samples from lung adenocarcinomas. We have also demonstrated that the pro-apoptotic effect of miR-125a-5p occurs through p53 upregulation. In summary, this report documents a close interaction between TIMP-1 and miR-125a-5p, with a clearly identified inverse correlation.

## MATERIALS AND METHODS

### Cell culture

NSCLC cell lines A549 and NCI-H460 were purchased from American Type Culture Collection (ATCC) and the cells were subcultured according to the recommended instructions. A549 cells were grown in F12-K media supplemented with 10% FBS and 0.1% Gentamycin. H460 cells were grown in RPMI-1640 media supplemented with 10% FBS, 100 μg/ml streptomycin, 100 units/ml Penicillin, 4.5 g/L glucose, 10 mM HEPES and 1mM sodium pyruvate. HEK 293T cells were grown in high glucose D-MEM media supplemented with 10% FBS, 100 μg/ml streptomycin, 100 units/ml Penicillin.

### Reagents and antibodies

Complete mini protease inhibitors and Phos stop (Roche, Mannheim, Germany) were prepared according to manufacturer’s protocol to be used with RIPA lysis buffer. The primary antibodies used are Anti-TIMP-1 (Millipore, California, USA), anti-Bcl-2, Bax, p-Akt (T308), Akt, p-mdm2 (Cell Signaling, Danvers, MA), p53, mdm2 (Santa Cruz) and anti-Actin from Sigma (St. Louis, MO). The primary antibodies were used at 1:1000 dilution unless mentioned otherwise. All primers were obtained from Integrated DNA Technologies (IDT). MiRNA primers were purchased from Exiqon (Woburn, MA).

### Short hairpin RNA (shRNA) transduction

Cells were transduced with TIMP-1 shRNA lentiviral particles targeting different TIMP-1 shRNA constructs (SIGMA) and a non-silencing control, as per the manufacturer’s instructions. Briefly, 2 × 10^5^ cells were seeded into 6 well plates and subsequently overlaid with a viral titer with multiplicity of infection (MOI) of 3 or MOI of 10. After 24 hours, the medium was replaced by standard culture medium. The cells were returned to the CO_2_ incubator at 37°C. Another 24 hours later, the cells were grown in a complete medium supplemented with puromycin (Gibco Life Technologies, 2 μg/mL) for selection. Successful shRNA integration was confirmed by downregulation of TIMP-1 protein. The sequences of shRNAs used for knockdown of TIMP-1 in this study are: 1) TRCN0000299345: 5′-CCGGGCACAGTGTTTCCCTGTTTATCTCGAGATAAACAGG GAAACACTGTGCTTTTTG-3′ (A549-KD1). 2) TRCN 0000303681: 5′-CCGGACAGACGGCCTTCTGCAATTCCTCGAGGAATTGCAGAAGGCCGTCTGTTTTTTG- 3′ (A549-KD2 and H460-KD).

### miRNA and plasmid transfections

Routine transfections were carried out according to established protocols either with Hiperfect (Qiagen, USA) or with Turbofect (Thermofisher, USA). Hiperfect was used for transfection of miRNAs or siRNAs and turbofect was used to transfect plasmids according to manufacturer’s protocol.

The miR-125a-5p mimics and antagomirs and the corresponding negative control (NC) were purchased from Exiqon (Woburn, MA). The mimics and inhibitors had Locked Nucleic Acid (LNA) technology to reduce off target effects and were fluorescently labeled to assess transfection efficiency.

A549 and H460 cells were seeded into 6-well plates at a density of 2 × 10^5^ cells/well and cultured at 37°C for 24 h. A final concentration of 10nM miR-125a-5p mimic, and 5nM miR-125a-5p inhibitor or NC was transfected into target cells using Hiperfect (Qiagen) and Opti-MEM^®^ I reduced serum medium (Thermo Fisher Scientific, Inc. USA), according to the manufacturer’s protocol.

### Rescue plasmids construction

Rescue plasmid was constructed to contain a CMV promoter, the full-length cDNA of TIMP-1 resistant to shRNA, and a Neomycin resistant gene. The shRNA binding site of TIMP-1 cDNA was mutated using PCR primers to create a shRNA resistant TIMP-1 cDNA. The mutated TIMP-1 expression cassette along with the selection marker was amplified by overlap PCR method from TIMP-1 expressing plasmid pCMV-TIMP-1 with the following primers: 1) pLL-Not-CMV: 5′-CAAGCTTAAGCGGCCGCTAGTTATTAATAGTAATCAATTACG-3′; 2) HTIMP-1-anti-KD2-R: 5′-GTCGGAATTGCAAAACGCAGTCTGTGGGTG-3′; 3) HTIMP-1-anti-KD2-F: 5′-CACCCACAGACTGCGTTTT GCAATTCCGAC-3′; 4) pLL-Eco-Neo-R: 5′-GTCCCTCGACGAATTCCTGGGACCGAACCCCGCGT-3′. The mutation sites are denoted by lower case letters. The amplified DNA fragments were subcloned into NotI and EcoRI site of pLentiLox3.7 plasmid to create pLL-anti-KD2—TIMP-1.The sequence was confirmed by DNA sequencing (MC lab, California). PLL-anti-KD2-TIMP-1 was transfected in A549KD2 cells according to the manufacturer’s protocol.

### Luciferase plasmid construct

A firefly luciferase reporter construct (psiCHECK) in which a fragment of 3′UTR or 5′UTR region of TIMP-1 was cloned, and transfected into HEK 293 cells. The TIMP-1 wild type 3′UTR region was cloned by using the published primers TIMP-1-F and TIMP-1-R [54] from A549 cDNA. The seed region was mutated by overlap extension PCR according to standard protocol. Using wild type TIMP-1 3′UTR sequence as the template, the down fragment was amplified by MutF2 and TIMP-1R primers. The up fragment was amplified by TIMP-1 Fand MutR2 primers to obtain the mutated products. Amplification of mutated products were done by MutF2 and MutR2 using the mutated up and down fragments as template. The first fragment was amplified by TIMP-1 F and R3 primers. The products of first fragment and mutated fragments were annealed (55°C) and extended (72°C) and gel purified and final mutated 3′UTR PCR was performed by TIMP-1F and Mut R2. The Xba I site was used to clone the UTRs in the psiCHECK plasmid and were transformed in competent DH5α cells.

For the HEK 293T cells, co-transfection with vehicle, an empty plasmid, or a control plasmid was performed to serve as controls. In addition, the constructs with mutated fragment of the 3′UTR of TIMP-1 without the putative binding sequences were used as mutated controls. The underlined primers are Xba I site added, the underlined and bolded segments are overlaps. The primers used are:TIMP-1F: 5′-CCCTCTAGAATCCTGCCCGGAGTGGAAGCTGAAGCC-3′; TIMP-1 R: 5′-GGGTCTAGATAAAAACCCAACATTTGGCATCCCTCAT-3′ 5′UTR-F: 5′-CCCTCTAGAGGTGGGTGGATGAGTAATGC-3′; 5′UTR-R: 5′-GGGTCTAGAATGGTGGGTTCTCTGGTGTC-3′. Primer R3: 5′-**ACTGGTCCCAGG**GTGCTCACACCAG-3′. MutF2: 5′-**CCTGGGACCAGT**TTTTTTTTTTTTTGC-3′. MutR2: 5′- GGGTCTAGATGGCATTTTTTTTTTTTACTGGTCC-3′.

### Micro-array analysis

GeneChip^®^ miRNA 2.0 microarrays from Affymetrix^®^ were used to examine the expression of 847 microRNAs in total RNA of A549 cells. The yield of RNA from 1–2 × 10^6^ cells of the cell lines ranged from 3–18 μg/μl. The hybridization was carried out at the Georgia Cancer Center facility according to manufacturer’s protocol. Chip images were evaluated for overall quality and hybridization quality was found to be consistent with the manufacturer’s requirements. The micro-array data analysis was done at the Georgia Cancer Center core facility.

### RT2 Profiler PCR array analysis

Primers were synthesized by the manufacturer (SA Bioscience, Frederick, MD). RNA samples were reverse transcribed using the RT^2^ First Strand Kit (SA Bioscience) according to the manufacturer’s protocol. PCR was performed using 25 μl of the following mixture in the wells of a 96-well microtiter plate (Bio-Rad): 1275 μl of 2× SuperArray RT2 qPCR Master Mix, 38 μl first-strand cDNA synthesis reaction, and 1435 μl of double-distilled nuclease-free water The temperature protocol included a start cycle for 10 min at 95°C, 40 cycles of amplification (15 s at 95°C, 15 s at 60°C, and 15 s at 72°C), followed by a melt curve. Thermal cycling and fluorescence detection were performed using a Bio-Rad iCycler (Bio-Rad). The Ct values of the target cDNAs were normalized by the average Ct of 6 housekeeping genes (GAPDH, ACTB, 18srRNA, GUSB, B2M and HPRT1) within the same 96-well microtiter plate. Negative controls (NTC, NRT) remained unamplified throughout the study. Calculations were conducted using a PCR array data analysis tool provided by the manufacturer.

### Quantitative RT PCR

RNA isolation was performed with the miRNeasy plus kit to obtain total RNA with efficient enrichment of < 200 nucleotide RNAs.

For mRNA: cDNA synthesis was performed with 1 μg of total RNA using the iScript RT supermix (BIORAD). The cDNA samples were diluted and amplified using the iTaq Universal SYBR green supermix in BIORAD CFX connect PCR machine. Primers used for PCR amplification were purchased from Genosys (TIMP-1F-AGCGCCCAGAGAGACACC; TIMP-1R-CCACTCCGGGCAGGATT).

For miRNA: cDNA synthesis was done by miRCURY LNA universal cDNA synthesis kit and miRCURY LNA SYBR green master mix (Exiqon, MA). The target sequence for hsa-miR-125a-5p is 5′-3′: UCCCUGAGACCCUUUAACCUGUGA.

The relative expression levels of target genes were analyzed by examining the mRNA expression of each target gene normalized to GAPDH and the miRNA was normalized to RNU6. Error bars represent Standard Error of Mean (SEM) of three independent experiments.

### Immunoblot assay

Cells were seeded in tissue culture dishes at a density of 3 × 10^4^ cells/cm^2^. Protein extracts were prepared using RIPA lysis buffer containing complete mini, phos-stop (Roche, USA) and PMSF (1 mM, Sigma). The protein concentration in each sample was determined by BCA Protein Assay kit (Pierce, Rockford, IL, USA). Proteins (40–60 μg) were separated by sodium dodecyl sulphate (SDS)–gel electrophoresis using 10–12% polyacrylamide gels and blotted on to PVDF membranes (Bio-RAD). The membranes were blocked in washing buffer (Tris-buffered saline (PBS) + 0.1% Tween 20) containing 5% dry milk or 5% Bovine serum albumin and incubated with the primary antibody. Subsequently, the blots were washed 3 × 10 min in washing buffer followed by incubation with the appropriate horseradish peroxidase-conjugated secondary antibody. Following 3 × 10 min washes in washing buffer, the blots were developed by the chemiluminescent detection system (Denville Scientific, NJ) according to the manufacturer’s instructions. In order to obtain a loading control, the blots were stripped and re-probed with a primary monoclonal antibody recognizing β-actin (SIGMA), diluted 1:10,000 in washing buffer containing 1% dry milk. Finally, the blots were washed 3 × 10 min in washing buffer and developed as described above.

### Flow cytometry

The cells were cultured for 24 hours and treated with 0.5 μM Staurosporine; after 1 hour the cells were trypsinized and washed with PBS+1% heat inactivated FBS (Gemini Biosciences) and resuspended in the 1X binding buffer provided in the kit (BD Biosciences). Cells were stained in Annexin V PE followed by the addition of 7AAD to detect dead cells and analyzed immediately in BD accuri c6 cytometer.

### Hoechst assay

Briefly A549 cells were grown for 24 hours in 6 well plates to a confluency of about 70–75%. The cells were transfected with either miR-125a-5p mimics (10 nM final concentration) or negative controls. After 48 hours the cells were washed with cold PBS and incubated with Hoechst 33342 (10 μg/ml) at 37°C with 5% CO_2_ for 15 minutes. Five different images of each well were taken immediately under fluorescence microscope using the DAPI filter (UV/488 dual excitation). The percentages of pyknotic or condensed nuclei were quantitated in controls vs treated samples in three independent experiments.

### Dual luciferase reporter assay

Dual luciferase assays were performed with the dual luciferase reporter assay system (Promega Corp., Madison, WI). The assay and the reagents were prepared according to the manufacturer’s protocol. Briefly, cells were grown to exponential phase in the appropriate medium, and 10 μl of cells was removed directly from culture and transferred to 100 μl of 1× passive lysis buffer. After allowing lysis for 10 to 15 s, a 10-μl aliquot was used for luminescence measurements with a Biotek synergy HTX multimode reader. The following steps were used for luminescence measurements: 100 μl of the firefly luciferase reagent (LARII) was added to the test sample, with a 10-s equilibration time and measurement of luminescence with a 10-s integration time, followed by addition of 100 μl of the *Renilla* luciferase reagent and firefly quenching (Stop & Glo), 10-s equilibration time, and measurement of luminescence with a 10-s integration time. The data are represented as the ratio of firefly to *Renilla* luciferase activity (Fluc/Rluc).

### Wound healing assay

Cell migration was determined by a scratch wound assay. For the scratch wound assay, 10^6^ A549 cells were plated in six-well plates and grown to confluency. Cells were consequently wounded with a sterile pipette tip to generate a cell-free gap of ~ 0.1-mm width, and the wound location in the culture dish was marked for exact location. Cells were washed with serum-free media and photographed using a Carl Zeiss Axiovert 200 camera to record the wound width at 0 h, and subsequently at 8, 24 and 48 hours.

### Cell invasion assay

The cell invasion assay kit (Chemicon, USA) was used to assess the invasive potential of cells. After incubation for 6 h at 37°C, cells passing through the EC matrix™-coated membrane onto the lower side of the chamber were fixed with 4% formaldehyde and stained with methylene blue. The migrated cells were then counted in 10 random fields at × 200 magnifications.

### Adhesion assay

Six-well plates were coated with Matrigel™ (10 μg/ml, Thermpfisher Scientific, USA), and 1% bovine serum albumin was used as the control. Cells were harvested with trypsin/EDTA and resuspended in serum-free medium. Cells were allowed to attach at 37°C for 1 h. Unbound cells were removed by washing twice with PBS. Attached cells were fixed in 4% paraformaldehyde and counted. Two individuals recorded cell attachment for these experiments. Cell counts were obtained by averaging the cell numbers from five wells.

### *In Situ* hybridization

Hsa-miR-125a-5p detection probe (3′ and 5′- end labelled with digoxigenin and LNA-modified) and miRCURY LNA™ microRNA ISH Optimization Kit 2 containing miR-125a-5p probe, U6 snRNA was used as positive control, scrambled miRNA LNA probe (as negative control) as well as miRNA ISH buffer were purchased from Exiqon (Denmark). The ISH procedure was adapted from the manufacturer’s protocol, and four micron FFPE sections were probed. For image acquisition, a photomicroscopy setup incorporating an Olympus BX40 microscope and SPOT Idea CMOS camera (SPOT Imaging Solutions, Sterling Heights, MI) was used. Images were assessed for overall increased or decreased expression, with an emphasis on determining histologic localization and distribution pattern of positive signal.

### Kaplan Meier curve

The Kaplan Meier curve was generated through the KM plotter, a web based software. The background database is manually curated. The database is handled by a PostgreSQL server, which integrates gene expression and clinical data simultaneously. To analyze the prognostic value of a particular gene, the patient samples are split into two groups according to various quantile expressions of the proposed biomarker. The two patient cohorts are compared by a Kaplan-Meier survival plot, and the hazard ratio with 95% confidence intervals and log rank *P* value are calculated. Each database is updated biannually (according to the website reference).

### Statistical analysis

Samples were plated/run in triplicate unless otherwise indicated, and all experiments were performed at least three times. Data represent mean and one representative experiment/image as shown. Generally statistical significance was determined by the two-tailed, non-paired Student *t* test, and data are reported significant as follows: ^*^, *P* < 0.05; ^**^, *P* < 0.01.
